# Rethinking networks through relatedness - a qualitative study among inpatients in forensic psychiatry

**DOI:** 10.3389/fpsyt.2026.1870584

**Published:** 2026-10-06

**Authors:** Berit Kjærside Nielsen, Lisbeth Uhrskov Sørensen, Caroline Trillingsgaard Mejdahl, Jette Møllerhøj, Morten Deleuran Terkildsen

**Affiliations:** 1 Public Health Research, DEFACTUM, Central Denmark Region, Aarhus, Denmark; 2 Department of Public Health, Health, Aarhus University, Aarhus, Denmark; 3 Department of Forensic Psychiatry, Aarhus University Hospital, Aarhus, Denmark; 4 Department of Clinical Medicine, Health, Aarhus University, Aarhus, Denmark; 5 Centre for Social Work, University College Absalon, Roskilde, Denmark

**Keywords:** forensic psychiatry, qualitative research, recovery, relatedness, social networks, sociograms

## Abstract

**Background:**

Recovery-oriented approaches in forensic psychiatry increasingly emphasize the importance of social relationships. However, research and practice have primarily approached significant relationships through a family-caregiver lens leaving less knowledge about the broader range of relationships that may be meaningful to patients. Drawing on the concept of relatedness, this study explores how forensic psychiatric inpatients understand and negotiate their networks of significant relations during long-term admission.

**Methods:**

A qualitative study was conducted in two medium secure forensic psychiatric units in Denmark. Twenty-four inpatients participated in semi-structured interviews supported by sociograms, which enabled participants to map and reflect on their social networks. Data were analyzed using a combination of descriptive analysis of sociograms and reflexive thematic analysis of interview transcripts.

**Results:**

Participants described their networks as fluid and context-dependent, encompassing not only biological relatives but also peers, former partners, professionals, and symbolically or digitally mediated relations. Three overarching themes were identified: (1) temporal trajectories, highlighting how relationships were shaped by past ruptures, present functions, and future hopes; (2) forms of relational presence, including emotional and symbolic connections as well as ambivalence; and (3) relational dynamics and agency, reflecting how participants actively maintained, redefined, or distanced themselves from relationships under institutional constraints. Relationships were characterized by both significance and fragility, with participants navigating tensions between connection, burden, and self-preservation.

**Conclusions:**

The findings challenge narrow conceptions of informal caregiving in forensic psychiatry by demonstrating that patients’ significant networks extend beyond family-based models. Adopting a relatedness perspective highlights the need for clinical practices that recognize diverse and patient-defined relationships, including symbolic and context-specific forms of connection. Tailoring involvement strategies to patients’ own relational priorities, while remaining attentive to ambivalence and potential burden, may support more nuanced and person-centered approaches to recovery in forensic psychiatric care.

## Introduction

Forensic psychiatry has increasingly turned toward recovery-oriented practices to improve rehabilitation (1, 2). Recovery is widely understood as a personal process of living a meaningful and satisfying life despite the limitations imposed by mental illness (3) with hope, autonomy, social inclusion, and personally valued goals as central principles (4, 5). Such processes may unfold through diverse and relationally grounded pathways, rather than solely through biomedical or risk-reductive approaches (6, 7).

Current trends in forensic psychiatric research and policy increasingly acknowledge the importance of involving informal caregivers as key figures in facilitating rehabilitation and recovery (8, 9). Involving informal caregivers supports medication adherence and reduces relapse risk, which may shorten hospital stays (10–12). However, compared to general psychiatry, forensic psychiatry unfolds within a care-and-custody framework at the intersection of mental health, law, and social justice, addressing individuals with complex health care needs (13, 14).

In Denmark, most forensic psychiatric patients are treated within general psychiatric services, while patients with more complex clinical and security needs may receive inpatient or outpatient treatment in regional medium-secure forensic psychiatric services, which are available across the five Danish regions (15). Placement in medium-secure care is based on an overall assessment of clinical needs, risk, and security requirements rather than the offense alone, although patients may have committed serious offenses, including aggravated assault or homicide. Care is provided by multidisciplinary teams within a physical and organizational environment designed to balance treatment, rehabilitation, and recovery with safety and security (14, 16). Admissions may extend over several years, depending on clinical progress, risk assessment, and legal circumstances. Visits are generally permitted but may be restricted following individual clinical and security assessments. Access to mobile phones and the internet is generally permitted but may be restricted when necessary for treatment or security reasons (17). These conditions, together with prior psychiatric trajectories, severe mental health problems, histories of criminal behavior, ambivalence toward illness, stigma, and safety concerns, can overwhelm patients and their families and pose substantial challenges to forming and sustaining meaningful relationships (18, 19).

Most studies of informal caregiving in forensic psychiatry have focused specifically on family caregivers. Qualitative studies have examined the experience of parents of forensic psychiatric patients (20, 21), while other studies have investigated the experience and involvement of family members more broadly (2, 8, 19, 22, 23). This family-oriented focus is also reflected in a recent qualitative evidence synthesis of 13 studies examining the experiences and involvement of family caregivers in forensic mental health care (9). While this literature provides important knowledge about family caregiving, considerably less is known about patients’ broader relational worlds: who they experience as significant, what these relationships mean to them, and how they are maintained and negotiated over time. To examine relational significance beyond relationships defined primarily through caregiving roles, we draw on Janet Carsten's (24) notion of relatedness. This perspective recognizes that significant relations may be grounded in genealogical and biological ties, but may also take more fluid forms shaped by changing circumstances and social contexts (25). It therefore provides a lens for examining how support, obligation, closeness, distance, and meaning are negotiated across different relationships within patients’ broader relational worlds. This study draws on the notion of relatedness to explore forensic psychiatric patients’ perspectives on their networks of significant relations during admission. Caregiving role, relational significance, supportive function, and preferences for involvement are treated as related but distinct dimensions of relational life, allowing exploration of who patients regard as significant, why these relationships matter, and how they are experienced within patients’ broader relational worlds. In doing so, the study seeks to contribute to a more nuanced understanding of the social worlds that may support or challenge recovery and inform relationally sensitive approaches to involvement in forensic psychiatric care.

## Methods

### Setting and recruitment

This qualitative study was conducted in two medium secure forensic psychiatric departments located in Central Denmark Region. The medium-secure forensic psychiatric services from which participants were recruited serve a population typically characterized by schizophrenia-spectrum and other psychotic disorders, high rates of co-occurring substance use problems, and histories of serious and/or violent offending (26, 27). Prior to recruitment, the first and last authors visited each department to inform both patients and staff about the study and its objectives. An easily understandable information sheet was displayed in common areas to support awareness and comprehension. To be included, participants had to be current inpatients, able to provide informed consent, not be in an acute psychotic state (as assessed by healthcare staff), and be able to speak and understand Danish. Participants were informed in both the written and oral study information that they would receive a bag of crisps and a soft drink after completing the interview. Individual-level information on psychiatric diagnoses, substance use disorders, and index offenses was not collected as part of the study, and the research team did not access participants’ clinical records. Obtaining such information would have required separate consent for record access or explicit inclusion of these topics in the research interviews. Given the study aim, the interviews instead prioritized participants’ experiences of significant relationships and relational life during admission.

### Data collection

Data were collected through semi-structured interviews. The interview guide, informed by a comprehensive literature review and consultations with healthcare staff, was designed to help identify individuals in the patient’s social network perceived as significant, regardless of the frequency of contact. Interviews were conducted by the second and last author: an experienced anthropologist and a physician with extensive clinical expertise in forensic psychiatry. The physician had no treatment responsibility for the interviewed patients and conducted interviews only at the hospital where she was not employed.

A sociogram was applied during the interviews to serve as a focal point and to visually represent the participants’ networks (28). Participants were asked to place significant others within the sociogram, arranging them according to their perceived closeness (from inner to outer circles) (**Figure 1**). Discussions focused on these placements, and participants adjusted their sociograms as they reflected on their relationships, moving some individuals closer or further away during the interview. This dynamic approach allowed for a deeper exploration of the participants’ perceptions of their relationships.

**Figure 1:**
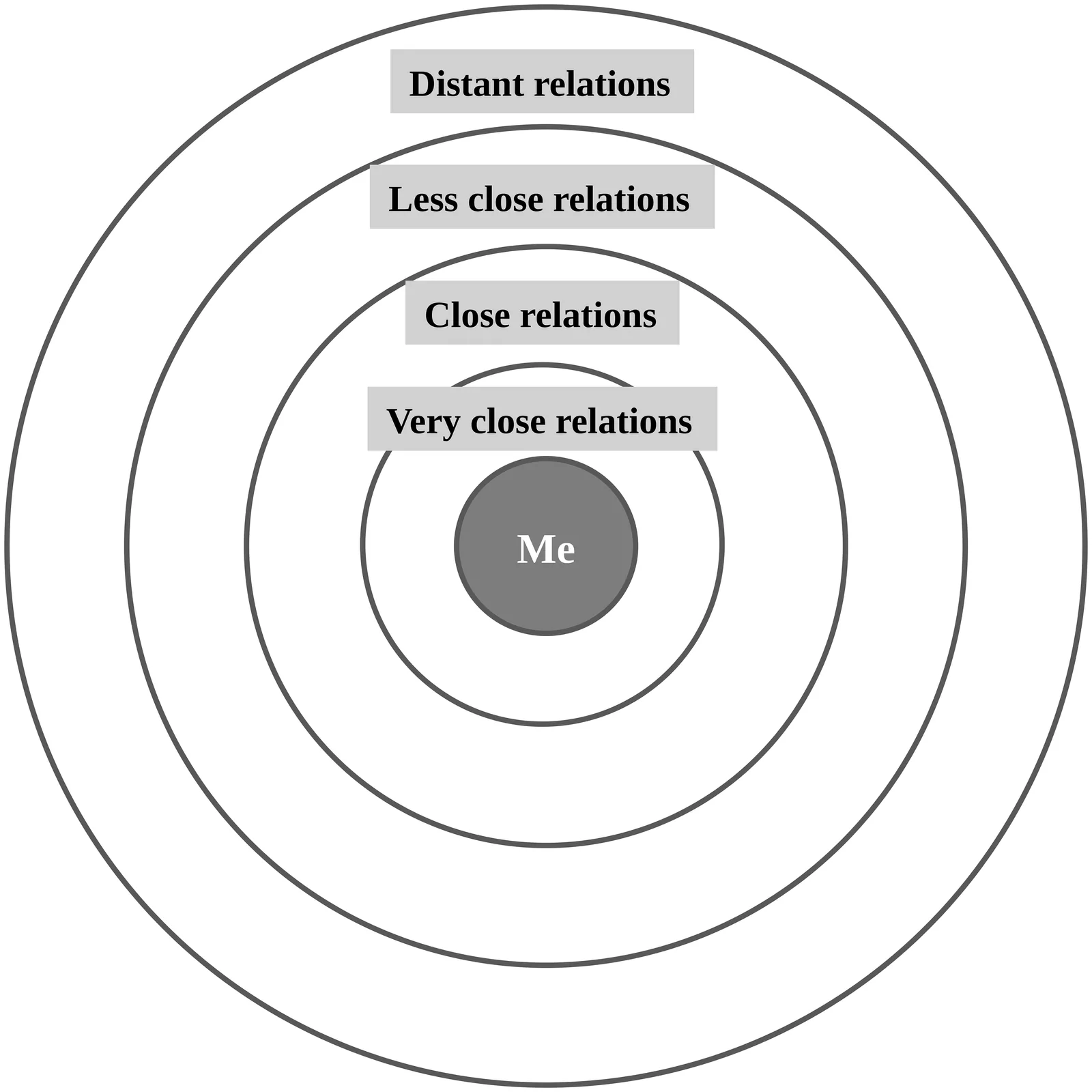
Sociogram used during interviews. Participants were asked to place individuals in their social network according to perceived closeness, with proximity to the centre indicating greater relational significance.

The interviews ranged from 10.5 to 62 minutes in length, with an average duration of 35 minutes. All interviews were audio-recorded with the participants’ consent and were subsequently transcribed verbatim by a student worker to ensure accuracy in data analysis.

### Data analysis

Data analysis involved both descriptive and thematic approaches. The sociograms were analyzed individually and collectively to quantify the number of identified network members, categorize the types of relationships, and assess their positioning within the sociogram, from very close to distant or outside the current network. This generated an overview of participants’ networks and offered insight into how relationships were prioritized and valued.

The interview transcripts were analyzed using reflexive thematic analysis (29, 30). The process was iterative and interpretive, encompassing familiarization with the data, systematic coding, the generation and refinement of candidate themes, and the development of analytic accounts. NVivo software supported data management. In line with Braun and Clarke’s reflexive orientation (30), coding and theme development were approached as active and situated processes shaped by the researchers’ perspectives. The interdisciplinary team discussed coding decisions and interpretive directions to enhance reflexivity and analytic depth. The combined use of sociograms and interviews made it possible to capture both structural features of participants’ networks and the lived meanings attached to their relationships, providing complementary perspectives on their social worlds. Special care was taken to ensure confidentiality in reporting. Given that only three women participated, all descriptions were phrased in gender-neutral and non-specific terms to prevent indirect identification.

## Results

The study included 24 participants, 21 men and 3 women, with an average age of 40 years (range: 26–55 years). Most had received a sentence to treatment around the age of 32 and had been hospitalized for a minimum of 4.5 years. Approximately 75% were of Danish origin. The interviews were shaped by varying levels of verbal fluency, reflection, and concentration among participants. Some responses were brief, which limited the extent of verbal elaboration in parts of the material. The sociogram played a central role in structuring the interviews, supporting participants in identifying and reflecting on significant relationships, and enabling discussion of relational meanings even when verbal elaboration was limited.

### Mapping networks of significance: descriptive patterns from sociograms

In total, 200 relationships were identified across the 24 participants’ sociograms. A synthesis of individual sociograms is presented in **Figure 2**, illustrating the distribution of relationships through the categories of closeness. Of the identified relationships, 47% were positioned as close, 20.5% as moderately close, 7.5% as less close, 7% as distant, and 18% as outside the participant’s current network. The distribution suggests that participants tended to position their relationships toward the closer end of the sociogram or outside their current network, with relatively fewer relationships occupying the immediate and distant categories. Importantly, placement reflected participants’ subjective experience of relational significance rather than frequency of contact. Individuals with little or no current contact, such as children, could therefore be positioned as close when experienced as emotionally significant.

**Figure 2:**
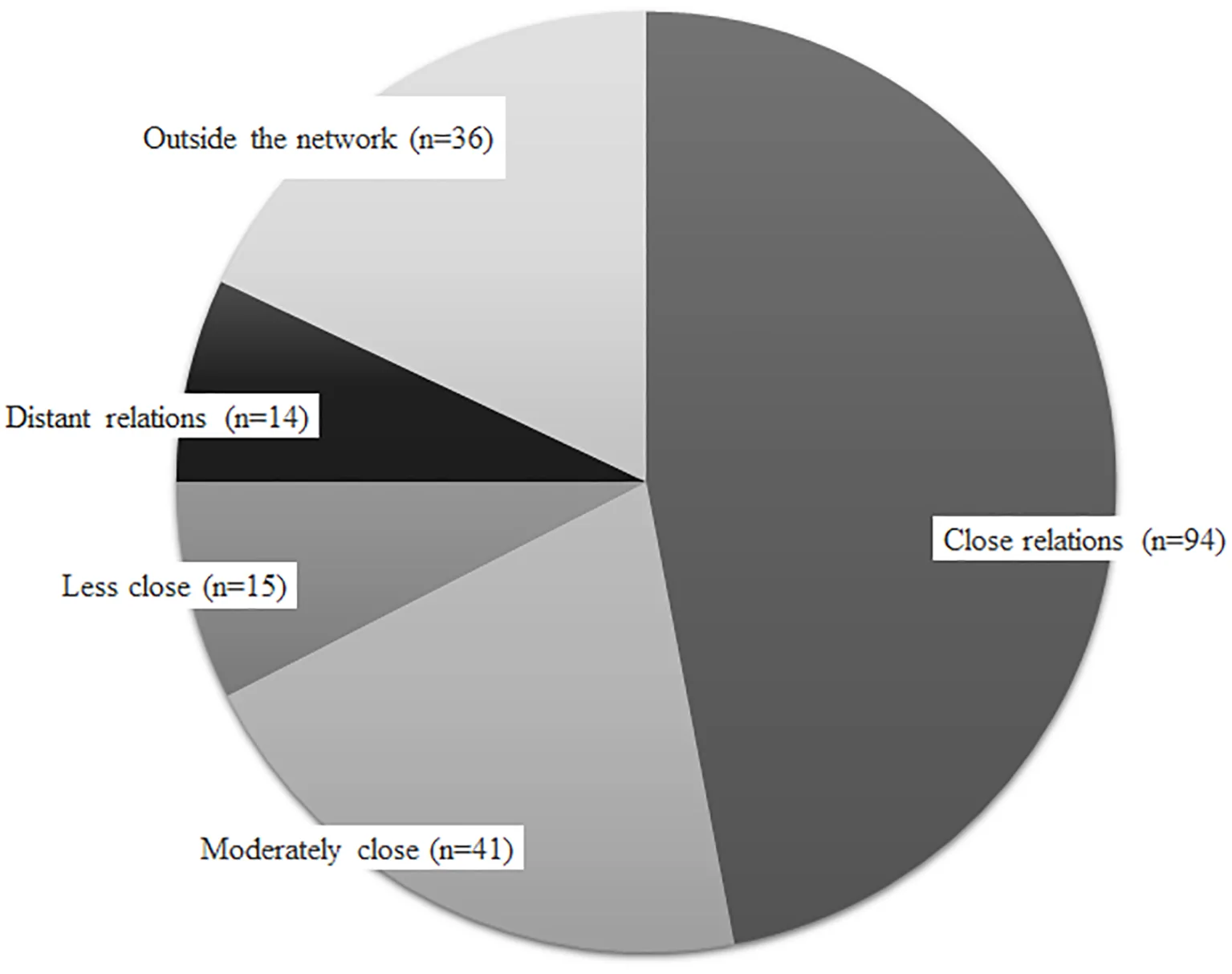
Distribution of relationships across categories. Combined sociograms from 24 forensic psychiatric patients (total relationships n = 200).

Parents were particularly prominent, with 16 participants placing a parent in the closest category, although parents appeared across all categories, ranging from daily to no contact for several years. Siblings and extended family were also frequently represented, with 12 participants placing siblings in the closest category. Friends and romantic partners were distributed across categories and were sometimes characterized by uncertainty about whether the relationship remained meaningful or active. Fellow patients were primarily situated within the closer categories, with seven participants placing a fellow patient in the closest category and ten in the moderately close category. These relationships were often embedded in the immediate institutional context and could be described as temporary or circumstantial. Staff were also prominent, with ten participants positioning staff within their closest networks, sometimes in friendship-like terms. Some participants expressed hope that supportive relationships formed during admission might continue after discharge. Finally, nine participants included an anticipated future close relationship in their sociograms, most commonly a future partner (n = 5), but also future friendships (n = 3), or animal companionship (n = 1).

### Experiencing and negotiating relatedness in forensic care: a thematic analysis

Reflexive thematic analysis revealed relationships and social networks as multi-layered and deeply contextual experiences for forensic psychiatric patients. Participants described how their connections, losses, and relational hopes affected their everyday lives, emotional well-being, and sense of self during long-term hospitalization. Three overarching themes were developed (**Figure 3**): (1) temporal trajectories (covering past relationships, present relations and functions, and future relational hopes), (2) forms of relational presence (encompassing emotional and symbolic presence, and contradictions and ambivalence), and (3) relational dynamics and agency (including maintaining and ending ties, navigating constraints, and autonomy and responsibility). While each theme is presented separately, they are interconnected, reflecting the complex interplay between institutional conditions, personal accounts, and future aspirations. In the following, each theme is explored in detail and illustrated with participant quotes to show how individuals navigate and make sense of their relational worlds.

**Figure 3:**
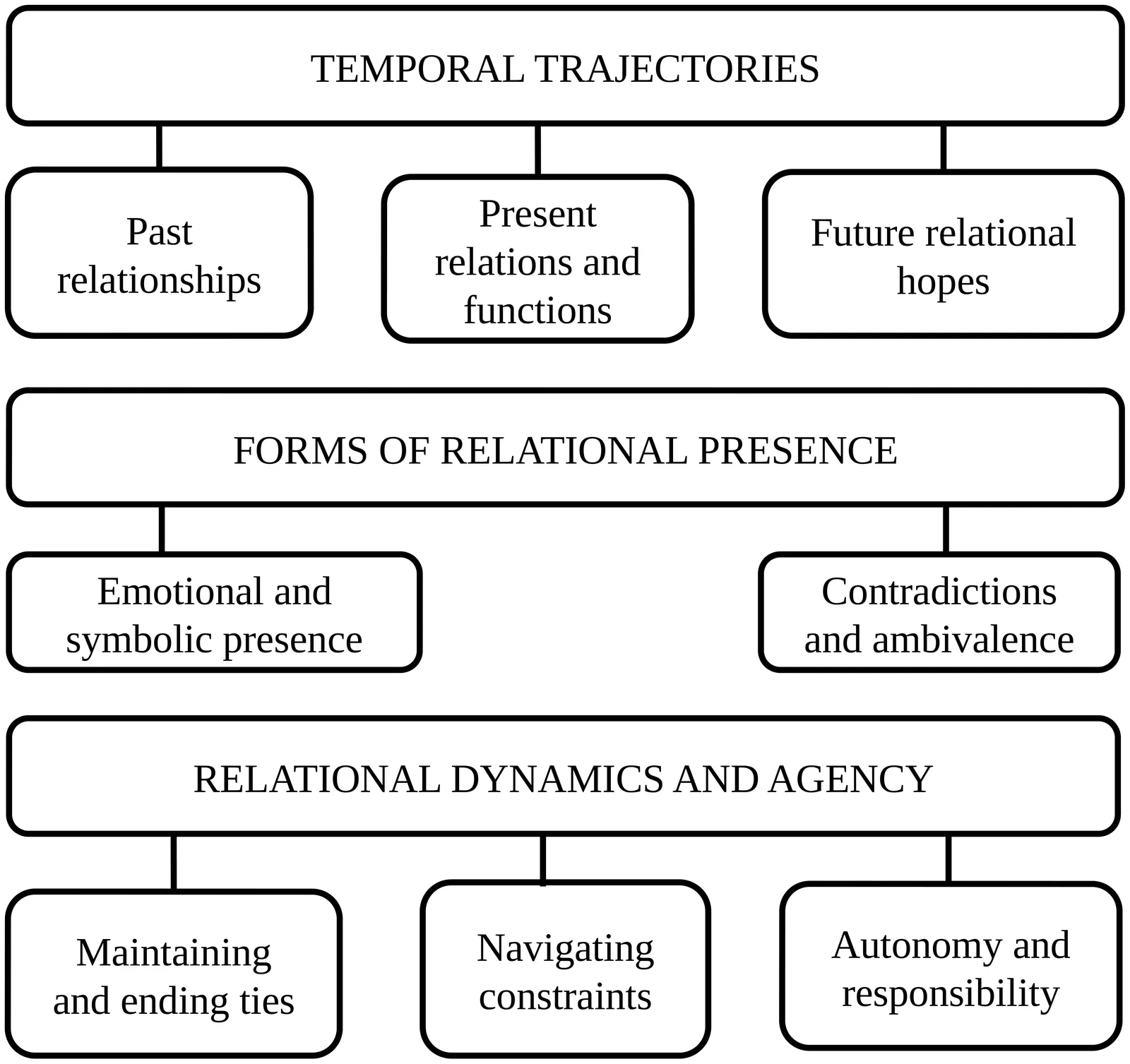
Thematic map of findings. The figure illustrates the three overraching themes and their associated subthemes, showing how participants’ experiences of relationships during forensic psychiatric admission were organized and interpreted in the analysis.

### Temporal trajectories

This theme captures how patients positioned their relationships across past, present, and future horizons. They described ruptures, ongoing ties, and hopes for reconnection, revealing how social bonds shape identity and coping during long-term hospitalization. Three sub-themes were developed.

#### Past relationships

Participants’ reflections on past relationships revealed how earlier ruptures and losses continued to shape their lives. Ties that had once been important often faded yet still carried emotional weight. One participant, when asked why a (specific) relationship ended, simply recalled:

“I don’t know what happened, I just became ill” (ID05),

framing the onset of illness as a sudden disruption that redefined the relational world. Others evoked memories of relationships that had once been positioned at the very center of their sociogram, described as “*in the green*”. One participant explained that a child and a sibling had been placed there when they were closest, but were now positioned at a distance, illustrating how the sociogram helped visualize shifts in relational proximity over time. This temporal repositioning revealed how relationships that had once been central continued to carry emotional significance despite absence (ID24). Several explained how friendships dissolved gradually during long admissions. Some described it as a form of withdrawal:

“others I never heard from again, they just chose to cut me off” (ID08),

emphasizing the sense of people that actively turned away. Others framed it as if relationships simply faded, friendships that had “*erased themselves*”, a subtle but painful form of social loss marked by silence rather than conflict (ID21), capturing the rawness of being silently left behind. Another participant reflected more ambivalently that “*friends just disappear*” but also reframed the reduction of relations positively:

“then you can start on a fresh” (ID24).

Others conveyed the pain of more abrupt splits. A participant shared that a sibling had decided the participant could no longer see their niece and nephew:

“I don’t feel so good about it, I’d like to see them again” (ID04).

Such statements revealed how absence in the present was narrated as a continuation of past rupture, where earlier closeness persisted as an enduring sense of loss. Yet, in their very incompleteness, these utterances also gestured forward, evoking a fragile horizon, where reunion, though uncertain, could still be imagined. In this way, the narratives held both mourning and momentum, hinting at a temporal thread through which hope persisted toward a future not yet foreclosed. Parental relationships held particular weight. One participant pointed out that

“the greatest loss I have is my children [ … ] no phone calls, no pictures, I don’t even know what they look like now” (ID18).

Despite this separation, hope remained that as the children matured, they might choose to reconnect, imagining the future as a time for reconciliation. For some, the rupture of ties was closely linked to the moral dimension of their offense. As one explained:

“they could not accept my offence … my children didn’t want contact. I could get some, but I don’t want to go against their wishes” (ID18).

Here, loss was experienced not only as situational but also as moral, as the participant held back out of respect for others’ boundaries despite their own pain. The account conveys a raw awareness that the possibility of future togetherness has been foreclosed, not by choice, but by a moral judgment that renders reconciliation impossible. In this sense, the participant’s restraint reflects recognition that the once shared world can no longer be reclaimed, leaving hope suspended in a space where others have already withdrawn. Others spoke of pushing people away deliberately as a protective strategy. One participant described having a biological child but choosing not to reach out:

“I don’t want them to know me here [ … ] and when I get out, it’s too late” (ID02).

In this account, withdrawal was described as more than avoidance. It reflects an attempt to pause one’s own relational life while confined, knowing that time outside continues to move on. The act of stepping back thus creates a temporal disjunction, a sense that while the participant waits in suspended time, relationships beyond the institution quietly slip away.

Others recounted former shared lives with partners and families “*out there*”, describing homes and routines that had collapsed under the weight of illness, bereavement, or substance use. These memories of closeness, ruptured family bonds, and fading friendships revealed how the past continued to shape present identities and future hopes, as participants narrated their relational trajectories with a mix of sadness, ambivalence, and hope.

#### Present relations and functions

Participants described their current relationships as key sources of stability and continuity in everyday life during hospitalization. For many, family members (most often parents) remained central figures, providing both emotional reassurance and practical support. One participant emphasized how relatives stayed in close contact, calling regularly, visiting, and joining meetings at the ward:

“They’ve been there for me in this difficult situation [ … ] they believe in me and they’re proud that I’ve changed” (ID23).

Such involvement offered tangible proof of care and belief in the participant’s recovery, reinforcing a sense of accountability and belonging. Others described relationships with parents as structured around mutual exchange, where giving and receiving gifts or money created a sense of reciprocity and normalcy. One participant explained how the family’s mutual generosity, sharing “*good things*” such as gifts, drinks, and everyday conversation, helped maintain closeness:

“We talk about how we’re doing, about the future … it’s really cozy” (ID24).

Though the conversations were often light, these exchanges sustained emotional connection and continuity with life outside the institution. For several participants, parents also functioned as primary communicative partners in navigating treatment and illness. One explained:

“It’s my parents who get to know the most about my treatment, they know how to handle schizophrenia” (ID06).

In such accounts, the family’s involvement was tied not only to affection but to shared understanding and experience, positioning relatives as knowledgeable allies in the management of illness.

Beyond family, everyday interactions with fellow patients and staff emerged as important relational anchors. These relationships were not described as intimate but as steady, low-pressure presences that structured daily life. As one participant put it,

“They’re not the ones you’re closest to, but they’re there. We watch films, play games, or just hang out” (ID16).

Such activities provided a rhythm of social engagement and moments of companionship within an otherwise controlled environment. For others, these ties carried a deeper emotional significance during admission, even if their durability beyond the ward was uncertain. One participant reflected:

“While I’m here, they’re close to me. I can talk with them about anything. But I don’t know what happens on the outside. This closeness belongs to being here” (ID18).

This ambivalence between genuine connection and awareness of its limits illustrates how present relations were both sustaining and contingent, embedded in the specific temporality of institutional life. Some also maintained external connections through digital or recreational activities. Online communities, for instance through gaming, allowed a sense of everyday sociality that felt “normal” and undemanding:

“They know I’m admitted, but we just play as if nothing happened and that’s fine with me” (ID12).

These ties provided continuity with pre-hospital life and moments of ordinary interaction that contrasted with the predictability of the ward. Finally, participants spoke about the comfort of faith-based or professional relationships that offered meaning and a sense of being understood, such as contact with a priest or trusted staff member.

Across these accounts, present relations served diverse yet interconnected functions. They provided emotional grounding, practical stability, and a sense of belonging within an otherwise restricted world. Whether maintained through daily family calls, shared routines with peers, or digital friendships, these connections upheld participants’ sense of self and orientation in time.

#### Future relational hopes

Across interviews, participants expressed forward-looking hopes that extended beyond the institution, revealing how the imagination of future relationships provided direction and meaning in the present. These hopes ranged from concrete plans for life after discharge to more tentative dreams of love, belonging, and reciprocity. Some described how relationships formed during admission had grown into genuine companionships they wished to preserve after leaving the ward. One participant spoke warmly of a peer with whom he shared daily routines and short outings:

“He’s a really good person, we spend time together, and I’d like to stay in touch when I’m discharged” (ID23).

Such accounts suggest that institutional ties, often seen as temporary, could evolve into lasting bonds that carried hope for continuity and a sense of shared recovery. Others articulated more uncertain hopes for intimacy and partnership.

“Maybe a nice girlfriend I could learn to live with”,

one participant reflected,

“but it’s hard to find someone when you’re in here [ … ] they might think you’re nice, but when you open up, they get scared” (ID23).

The wish for ordinary love was shadowed by anticipated rejection, showing how stigma shaped the horizon of relational possibility. Hopes for closeness were therefore both resilient and fragile, grounded in longing yet marked by fear of exclusion. For several participants, imagining the future also meant envisioning forms of connection beyond romantic or family ties. One described a wish to engage in voluntary work upon release, as a way to “*give something back*” and transform experience into contribution:

“Maybe at a drop-in centre or Red Cross [ … ] it’s given me a new perspective on things” (ID18).

Though the participant acknowledged the sadness of receiving early retirement,

“I hadn’t seen myself as a pensioner”,

the idea of volunteering offered a way to restore dignity and relatedness, to participate again in the moral world of others. Hopes for work and everyday normalcy were similarly expressed as aspirations for reintegration and agency:

“I hope to get a job, maybe live a normal life,”

one participant explained, adding that these conversations took place mainly with his family rather than staff:

“We talk about where things are heading, what might happen one day” (ID22).

The family appeared as a relational space where the future could still be imagined, in contrast to institutional routines that left little room for personal projection.

Some participants linked their hopes explicitly to care and companionship rather than independence.

“I hope to move into a sheltered living place, to be among people, especially staff,” a participant said (ID13).

Their vision of wellbeing rested not on autonomy but on sustained social presence, an image of recovery defined through proximity rather than separation. Others held onto distant but meaningful hopes of reconnection with estranged family. One participant planned to write to their oldest child once they turned 18:

“I’ve tried through their mother, but she doesn’t reply [ … ] it’s sad, but I’ll try again when he’s old enough” (ID50).

Here, the threshold of adulthood became a symbolic opening, a future moment when communication might again be possible. Such hopes, however delayed, kept alive a sense of relational direction amid waiting. Future-oriented imagination also included non-human forms of companionship:

“When I move, I’ll get two cats again … they make me happy … they’ll be part of my network”, one participant said (ID10).

In this reflection, the wish for future pets symbolized emotional continuity and belonging, a way of reclaiming ordinary roles and identities, “*they’ll make me a cat dad*”. Finally, future hopes could take shape through imagined togetherness. One participant described in detail the apartment he and his partner had rented for after his discharge:

“We already have a flat. I’ve never sat on the sofa or in my new bed [ … ] it’s strange, but I’m okay with that” (ID02).

The home, though unseen, functioned as a symbolic anchor, a projected site of return and relational restoration, sustained through loyalty and imagination while waiting.

Across these narratives, future hopes wavered between the concrete and the impossible, between ordinary aspirations and the awareness of how time and stigma reshape what can be hoped for. They revealed a capacity to orient toward life beyond the institution, even as the boundaries of that life remained uncertain. Hope thus appeared not as naïve optimism but as a quiet insistence, a way of remaining in relation to imagined others, and of keeping the future open, however precariously.

### Forms of relational presence

The theme encompasses the ways in which participants describe closeness, connection, and absence. This includes both emotional and symbolic presence, relationships maintained through thought, prayer, or memory, as well as contradictions and ambivalence, where closeness and distance coexist. Participants often articulated the significance of feeling connected even when physical contact was limited or impossible, and they navigated tensions between longing, loyalty, and disillusionment. Two sub-themes were developed.

#### Emotional and symbolic presence

Participants described forms of presence that exceeded physical co-location, showing how connection could be sustained through faith, memory, routine, and small embodied gestures. Spiritual practice figured as a salient mode of relatedness to something beyond the ward, one participant noted praying every day and experiencing divine help through difficulties. Family ties likewise operated as enduring anchors of worth and recognition. A participant emphasized ongoing love and pride from relatives, continued contact “*even though I got into this*” and satisfaction that substance use had ceased, framing family as a source of stability and moral affirmation (ID23). Embodied contact, when available, took on heightened significance. Weekly hugs from a parent were described as “*keeping reality*” and countering a state of being

“skin-hungry [ … ] if I didn’t have them, I would go mad” (ID18).

In this case, touch was not merely affection, it functioned as a rare, regulating connection that affirmed personhood within an otherwise touch-deprived setting. The same participant explained that contact with people outside “*kept identity intact*”, preventing a slide into self-doubt: without such ties, one

“wouldn’t know who [one] was anymore” (ID18).

In this way, relational presence was read as protection against moral and psychological erosion. Not all emotionally meaningful ties were expected ones. A participant described a lawyer’s letters or simple notes such as “*take care of yourself*”, as expressions of care stronger than those from family (ID21). Here, empathy and trust were conveyed through writing, illustrating how intimacy can emerge in unexpected relational contexts. Healthcare staff were sometimes described as “*family-like*”, reflecting their central role in everyday life. Yet participants also recognized that this kind of family had to change with each transfer, a closeness shaped by necessity rather than lasting permanence (ID13). Moments of distress revealed the same dynamic: when a participant broke down after a sibling’s death, staff

“came in, talked to me, and calmed me”,

approximating the responsiveness typically reserved for intimate ties (ID13).

Within the ward, peers offered a distinctive kind of presence grounded in shared experience. One participant explained that fellow patients could

“talk about everything that happens on the unit”,

including episodes outsiders could not see. A peer’s messages, “*hold on*”, helped de-escalate a crisis and reassured both the participant and staff outside the door (ID25). Frequency of contact also carried symbolic weight. A friend who visited monthly was placed closer in the sociogram precisely because regular presence signified commitment (ID06). Under restrictions, participants crafted digital and ritual substitutes for co-presence: daily video calls with a partner, “*sleeping together*” via the phone and exchanging goodnight gestures, enacted a powerful sense of intimacy despite years apart (ID02). Similarly, weekly shared prayer over the phone created a felt togetherness when church leave was not possible (ID01).

Across these accounts, emotional and symbolic presence was shown to sustain identity, regulate affect, and affirm worth under conditions of limited access and high stigma. Presence may be embodied (a hug), enacted through routine (regular calls or visits), or mediated (letters, video calls, prayer by phone), what matters analytically is the recognition conveyed. These practices render participants visible to significant others and to themselves, preserving a fragile continuity of self in a context where much else falls away.

#### Contradictions and ambivalence

Participants’ accounts often revealed tension and ambivalence in how relationships were experienced. Feelings of closeness and distance frequently coexisted, shaped by the contradictions of living within an institutional environment where care, control, and dependency intertwine. Several participants described how confinement clarified who truly remained present in their lives.

“You find out who your real friends are when you’re in here”, one said briefly (ID05).

Such reflections carried both acceptance and resignation, a recognition of loyalty, but also the pain of exclusion. These mixed feelings were particularly evident in descriptions of interactions with staff. Some participants expressed warmth and trust while simultaneously emphasizing professional boundaries: “*It’s tricky*”, a participant explained,

“because I know they’re at work. They have a job to do [ … ] it’s not quite the same for me” (ID22).

(ID22). Others echoed this ambivalence in the sociogram exercise, hesitating where to place a staff member:

“She’s just an employee [ … ] maybe she’s just doing her job” (ID03).

In these descriptions, participants balanced gratitude for care with awareness of its institutional conditions, a relationship marked by both authenticity and constraint. Some participants highlighted subtle differences among staff, distinguishing those who disclosed small personal details or emotions from those who maintained strict professionalism. As one put it:

“The ones who tell me about their own life, that makes a difference [ … ] it feels more equal” (ID11).

Here, professional distance was not rejected but negotiated, and equality was felt when reciprocity was possible, even within asymmetrical roles. Ambivalence also characterized relationships with people outside the institution. One participant feared being perceived as dangerous by family members who avoided speaking about the offense:

“They’ve parked it somewhere,”

he said,

“and I’d like to show them that things are getting better” (ID25).

Another reflected on friends from before admission:

“One just became a father [ … ] he was very close to my children, and I imagine I would have had a similar role in his family. But I try not to ask too much about the real world” (ID18).

These examples capture how maintaining contact with the outside can evoke both comfort and pain, a connection that keeps one oriented to life beyond, yet reminds one of separation. Inside the institution, participants also drew boundaries between “*here*” and “*out there*”, describing two distinct social worlds that could not easily overlap. “*In here is my world*”, one explained.

“That world out there, I’m not part of it” (ID02).

The awareness of inhabiting parallel worlds produced both safety and loss: connection within the ward could feel genuine, yet at the cost of disengagement from the wider social world.

Together, these accounts show that relational life in forensic settings is seldom stable or singular. It is lived through contradictions, between professional duty and personal care, between gratitude and mistrust, between belonging and exile. Ambivalence itself becomes a mode of adaptation. A way of sustaining connection while protecting oneself from the pain of its possible withdrawal.

### Relational dynamics and agency

The theme depicts the processes through which participants maintain, adjust, or terminate relationships, and the extent to which they feel empowered or constrained in doing so. Participants spoke of making deliberate choices to strengthen or end ties based on trust/distrust, while also describing institutional limitations and external factors shaping their networks. The theme highlights how autonomy, responsibility, and vulnerability intersect in the management of relational life within the context of forensic psychiatric care. Three sub-themes were developed.

#### Maintaining and ending ties

Participants spoke about the active work of maintaining, adjusting, or ending relationships as part of their ongoing efforts to regain control and integrity in their social worlds. Choices about connection and distance were rarely simple, they carried moral, emotional, and practical weight, and were often influenced by the conditions of forensic care. Several participants described intentionally ending ties with people associated with substance use or crime to support their own recovery:

“I wanted to get away from the drugs [ … ] many of my old friends still use and live in that world, so I decided to let them go” (ID23).

Breaking contact was difficult but framed as an act of necessity. Others maintained relationships despite tension or sacrifice. One participant described how their father’s decision to stay in contact caused conflict within the family:

“He lost contact with my siblings because he wanted to see me. They couldn’t accept that he had contact with me” (ID18).

The participant expressed gratitude and a sense of debt:

“I owe him for what he’s done for me”.

Here, continued closeness was experienced as both comfort and obligation, a tie sustained through loyalty and repayment. A few participants described choosing to end contact with family altogether.

“I have no contact with my family, that’s how I prefer it”,

one said.

“They tried to reach out, but I said no. It was me who broke the connection” (ID13).

For this participant, separation was not loss but protection, a way to maintain psychological boundaries after a history of strain. Other accounts revealed more ambivalent forms of distance. Some acknowledged the difficulty of sustaining relationships shaped by past offenses or stigma:

“I tried with a few people, but my crime was too heavy, it became too difficult” (ID11).

Another participant described deliberately keeping “*a certain distance*” between the institution and the world outside:

“That’s why I don’t call it home, I want to keep them separate” (ID18).

Distance thus functioned both as self-care and as recognition of the gap between institutional life and the outside world. Even when relationships were maintained, they required continual negotiation. One participant spoke of a friend rarely seen during admission but still imagined as part of a future life:

’I haven’t invited him yet, but when I’m settled in my house, I’ll invite him to a house-warming party” (ID06).

This small gesture signaled a wish to reconnect and transform waiting into anticipation. Finally, several participants described selective closeness within the ward itself. One explained,

“I don’t feel close to everyone here. They each have their reasons for being here, and so do I” (ID13).

Here, choosing not to get too close to peers showed both reflection and awareness of the institutional context, suggesting that connection was a matter of choice rather than circumstance.

Across these accounts, maintaining or ending ties appeared as an ongoing practice of moral and emotional regulation. Participants acted as agents within constraint, weighing loyalty against self-preservation, care against risk. The capacity to choose, even to step back, became a form of agency, allowing participants to shape their relational worlds within and beyond the boundaries of institutional life.

#### Navigating constraints

Participants described how institutional life set the terms for when and how relationships could unfold. Staff routines and the constant presence of professionals shaped the atmosphere on the wards and could limit spontaneous interaction between patients.

“There’s always staff around when we sit in the common room”

one explained.

“Maybe that’s why it’s harder to talk” (ID22).

The presence of staff could thus shape not only the spaces available for social interaction but also participants’ sense of privacy and what could comfortably be shared with peers. At the same time, professional relationships could provide a different form of relational space. For some participants, staff were the preferred confidants for treatment-related or personal matters precisely because of their professional role and expertise. One explained,

“I wouldn’t want my family involved in my treatment. I’d rather talk to the professionals here, they’re trained to handle this, they understand” (ID23).

Professional presence could therefore be experienced both as a constraint on informal social interaction and as a valued source of understanding and support. During the sociogram exercise, a participant positioned their daughter in the outer circle, explaining,

“I’m not allowed to talk to her or see her, not while I’m admitted” (ID12).

When asked why she remained important, they simply replied, “*She’s my daughter*”. This brief exchange captures both the emotional persistence of family bonds and the institutional restrictions that render them inaccessible. The sociogram here served as a visual record of constrained relatedness. For some, the emotional cost of maintaining outside relationships was too high. One participant declined visits entirely, including from a partner:

“I don’t want anyone to visit me [ … ] I’m not ashamed of psychiatry, but I have a hard time saying goodbye. You must be in one world at a time, so you don’t break down too much” (ID02).

This expression of self-protection shows how separation could be both painful and necessary. Others described how institutional isolation gradually altered their sense of belonging.

“You live a bit inside a bubble in here, you’re not really part of society”, one participant reflected (ID08).

The passage conveys how the boundaries between “inside” and “outside” worlds can harden over time, producing distance not only from others but also from social imagination itself. Finally, participants recognized that even structured support carried its own restrictions. One mentioned that their legal guardian visited every few months to review progress and court matters (ID06), while another observed that rules governed staff availability and shared activities:

“It depends on whether they have time or staffing, there are even rules for how often we can play games or watch films” (ID07).

These examples highlight how institutional logics enter social life, defining when care is possible and when it must wait.

Across participants, navigating constraints meant learning to live within a system where connection was monitored, timed, and conditional. Participants developed small strategies to preserve dignity and coherence, keeping some worlds separate, limiting expectations, or finding comfort in predictability. Relationships were thus lived within boundaries that were not only spatial but moral and emotional, reflecting how institutional care both enables and restricts the experience of being with others.

#### Autonomy and responsibility

Participants frequently described the tension between maintaining autonomy and fulfilling the responsibilities they felt toward their families, staff, and themselves. Many expressed a desire for privacy, particularly regarding personal issues or treatment, highlighting the tension between self-protection and emotional transparency. One participant explained their desire to keep certain aspects of their life private, saying,

“I don’t want them involved in this, I want to keep it to myself and my therapist” (ID23).

This desire to shield family members from the emotional burden of their illness reflected a need for autonomy and control over what is shared. The participant continued,

“I’ll tell them if it really gets bad, but otherwise I want to keep it private”.

This decision underscores the emotional cost of vulnerability and the participant’s desire to maintain a sense of normalcy in familial relationships. Another participant described the complexity of involving family in their treatment:

“I couldn’t think of involving my mother in my treatment, then it would be like making her my guardian, and I’m an adult” (ID05).

Here, the participant emphasized the importance of autonomy within family dynamics, where even well-intended involvement was seen as potentially infantilizing. This suggests a clear boundary between personal agency and familial care, with the participant asserting their independence despite the emotional closeness of their mother. In contrast, one participant spoke about how they had carefully selected the friends and relationships they wished to maintain, stating,

“I don’t need any more people in my life right now. I have my parents and a couple of good friends, and that’s enough” (ID24).

This decision echoes the participant’s sense of responsibility to maintain a small but meaningful social circle, balancing the need for connection with the desire for emotional stability. Participants also reflected on how familial expectations influenced their sense of responsibility. One explained:

“They expect me to have a somewhat normal life, to stay free from substance abuse, that’s really what it all stands on” (ID16).

Here, the participant recognized the pressure of meeting family expectations, particularly regarding recovery, while also accepting the limitations that institutional life placed on their autonomy. Finally, another participant described the difficulty of maintaining autonomy in familial relationships, stating,

“I don’t want them involved in this … they don’t understand” (ID03).

Limiting family involvement appeared to reflect both a perceived lack of understanding and a wish to retain control over what was shared. Rather than indicating disengagement from the relationship itself, the account illustrates how relational closeness and preferences for involvement may diverge, and how maintaining autonomy can involve setting boundaries around disclosure and participation in treatment.

## Discussion

This study explored how forensic psychiatric inpatients understand, sustain, and reshape relationships during long-term hospitalization using sociograms and in-depth interviews. Across the material, significant relationships emerged as dynamic and negotiated forms of relatedness shaped by institutional conditions, personal histories, and anticipated futures. The findings extend research on recovery in secure settings by foregrounding patients’ own perspectives on which ties matter and why, while providing a broader relational context within which caregiving roles and preferences for involvement can be understood.

A central contribution of this study is to situate caregiving within patients’ broader relational worlds by showing how caregiving roles, supportive functions, relational significance, and preferences for involvement may overlap without necessarily coinciding. Existing research has documented challenges faced by family members, including stigma, fear related to the offense, emotional strain, and limited understanding of forensic trajectories (8, 20, 21). Our findings add a different perspective by showing that patients identified a wider range of significant relations, including peers, former partners, digitally mediated contacts, professionals, and symbolically present relations alongside biological relatives. Consistent with Carsten (24) notion of relatedness, significance was shaped not only by kinship or supportive function, but also by recognition, shared history, everyday interaction, symbolic presence, and hoped-for future connection. Importantly, findings from general psychiatric settings suggest that involvement of relatives cannot be assumed to be unproblematic or uniformly supportive. In a recent study of informal caregivers in general psychiatry, more than half of relatives reported experiencing high or very high levels of burden, spanning emotional strain, disruption of everyday life, and challenges in collaboration with mental health services (31). Qualitative research among relatives of people with severe mental illness similarly illustrates the duality of caregiving, where love and hope coexist with substantial responsibility, self-sacrifice, and a need for respite and recovery (32). Against this backdrop, a broader understanding of patients’ relational worlds should not be taken as an argument for extending involvement to a wider range of relationships. Rather, it highlights the need to distinguish relational significance from caregiving function and to consider which relationships patients wish to involve, how, and with what potential benefits or burdens for those involved.

The emergence of heterogeneous networks can be understood in relation to the restrictive nature of long-term secure care. Institutionalization disrupts ordinary social roles and can erode existing ties over time (6, 33–35). Yet participants were not simply cut off from previous social worlds. They actively negotiated whom to remain connected to, whom to distance themselves from, and which ties were too emotionally demanding to sustain during admission. Some limited contact to protect loved ones from worry or from exposure to the forensic environment, echoing accounts of relational strain and moral burden across long forensic trajectories (19). This resonates with research outside forensic psychiatry showing that people with severe mental illness actively shape their social worlds, including through negotiating distance from others and spending periods of time alone (36). From this perspective, reduced contact or a smaller network should not necessarily be understood as relational deficit. Rather, distancing and ending ties may, alongside efforts to maintain valued connections, form part of relational agency. Viewed in relation to recovery, this suggests that relational agency may involve not only establishing and sustaining connections, but also determining which relationships to maintain, redefine, limit, or end. In forensic psychiatry, however, such agency is exercised within institutional conditions that simultaneously shape opportunities for contact, privacy, and participation in social life.

Patients’ relational realities may sit uneasily with how involvement is institutionally recognized and organized. Hörberg et al. (37) show that staff often orient involvement toward “ordinary families”, that is, biologically and legally defined relatives who fit familiar narratives of care and responsibility, while confidentiality is frequently described by professionals as a key barrier to involvement in forensic psychiatric care (38). Our findings add a patient perspective by showing that familial or formal status does not necessarily correspond to relational significance or to patients’ preferences for involvement. Some biological relatives remained highly significant, but were explicitly excluded from treatment-related involvement, whereas relationships outside conventional family categories could be meaningful without necessarily being appropriate for formal involvement. Participants thus actively negotiated who should be involved, how, and for what purposes, including concerns about burdening relatives, destabilizing fragile ties, or disclosing information about illness or offense. These concerns echo research on stigma, fear of harm, and moral responsibility in forensic caregiving (8, 19). From a relatedness perspective, selective involvement may therefore reflect relational boundary work through which patients negotiate closeness, disclosure, protection, and self-preservation.

Participants also described staff as “family-like” or as stable everyday companions, and staff were sometimes placed in the innermost circles of sociograms because of consistent presence and perceived trustworthiness. This aligns with research emphasizing the importance of everyday staff–patient relationships for safety, stability, and recovery in long-term secure settings (7, 33, 39). However, participants’ accounts also revealed an inherent ambiguity in these relationships. The same professionals who provided companionship, emotional support, and spaces for disclosure were embedded within institutional structures of regulation, surveillance, and risk management. Staff presence could therefore facilitate relational security while simultaneously limiting privacy and spontaneous interaction with others. Participants appeared acutely aware of this dual position, sometimes questioning whether experienced closeness reflected genuine reciprocity or professional obligation. Read through relatedness, staff–patient relationships were therefore neither straightforwardly professional nor equivalent to informal relationships. Rather, their significance emerged within an asymmetrical institutional relationship in which closeness, care, authority, and constraint could coexist.

A further contribution concerns the importance of emotional and symbolic presence in sustaining relatedness under restriction. This resonates with social understandings of recovery that emphasize that recovery is not solely an individual process but unfolds through relationships and within particular social contexts (40, 41). Participants described connections that persisted despite physical distance, including memories of former partners, imagined futures with children, and digital or spiritual practices that created a sense of being accompanied. Such presence functioned as an anchor of identity and recognition rather than simply optimism or escape. The recovery relevance of relationships may therefore lie not only in the provision of practical or emotional support, but also in sustaining continuity, belonging, recognition, and a sense of connection to social worlds beyond the institution. Meaningful connection under forensic conditions may thus be episodic, mediated, or symbolic rather than dependent on physical proximity or regular interaction.

Peer relations similarly showed forms of support that were meaningful in everyday life without necessarily corresponding to formal caregiving or involvement roles. This resonates with recent research on forensic peer support, which highlights shared experience, trust, mutuality, and recognition as distinctive relational qualities through which peers may support connectedness and recovery processes (42). Participants described companionship, shared understanding, and moments of mutual regulation during distress, but differed in whether they wished to maintain peer relationships after discharge. This variation highlights the context-dependent and ambivalent nature of peer relations: they may provide meaningful everyday support during admission without necessarily being experienced as relationships that should extend beyond the institutional setting.

Finally, future-oriented hopes emerged as an important thread. Participants imagined reconnections with children, shared homes with partners, or the companionship of animals. Although often tentative, these hopes provided direction and coherence over time, consistent with recovery-oriented research in long-term secure care (6, 34). From a relatedness perspective, these narratives show how connection extends forward as an anticipatory practice even when present networks are limited.

Viewed from a recovery perspective, these findings caution against equating relationally oriented care with maximizing social contact or involving more people in treatment. Clinical practice should instead systematically explore patients’ own relational networks and preferences as part of assessment and care planning, rather than relying on predefined categories of family involvement. Attention should include symbolic, distant, or digitally mediated connections where these are meaningful to patients, while decisions about active involvement should be guided separately by patients’ preferences, the function and quality of the relationship, and potential benefits or burdens for those involved. Relational practice should therefore support valued connections while also legitimizing patients’ efforts to redefine relationships, set boundaries, or disengage from ties they experience as detrimental. This study has several strengths. The relatively large sample for qualitative research, particularly in a forensic setting, and the inclusion of two inpatient sites broaden the scope of the results and strengthen their relevance across similar institutional contexts. The combined use of sociograms and semi-structured interviews offered complementary perspectives on both network structures and the lived meanings attached to relationships. The sociogram provided a concrete reference point that supported recall and sustained focus among participants with varying levels of verbal fluency, while allowing participants to position and reposition significant relationships. In this way, it helped make the fluidity of relatedness visible and facilitated access to relational meanings that might not have emerged through verbal interview alone. These strengths should be considered alongside several limitations. Participants who agreed to take part generally appeared to have at least some existing social connections, suggesting that individuals with very limited or fragmented networks may be underrepresented. In addition, recruitment within a clinical setting may have been shaped by staff assessments of patients’ capacity to participate, potentially influencing which perspectives were included. The involvement of a clinician as one of the interviewers, although carefully managed and discussed within the research team, may have influenced how openly some participants chose to disclose sensitive experiences. Furthermore, the findings are situated within Danish medium secure forensic psychiatric services and reflect the specific institutional, legal, and cultural conditions of this context. While this supports in-depth understanding, it may limit transferability to other forensic systems with different organizational structures or patient populations. Finally, communicative constraints, including brief responses and fluctuating concentration, limited the depth of some narratives. While the sociogram mitigated these challenges by offering concrete entry points, its structured format may also have subtly guided how relationships were positioned and described. In line with a reflexive thematic approach (30), the analysis should be understood as an interpretive process shaped by the researchers’ perspectives and disciplinary backgrounds, which may have influenced both the development of themes and the emphasis placed on particular aspects of the data.

## Conclusion

This study demonstrates that forensic psychiatric patients’ relational worlds encompass a broad range of significant relationships that cannot be reduced to biological kinship, caregiving roles, or formal institutional categories. Patients’ relational worlds are shaped by shifting histories, constraints, aspirations, and emotional needs, resulting in configurations that are fluid rather than fixed. Approaching these networks through a relatedness perspective highlights how significance is experienced, negotiated, and sustained under conditions of long-term admission. Such an orientation offers a patient-centered lens for understanding which relationships matter and why, while distinguishing relational significance from preferences for involvement in care. Supporting patients in maintaining, redefining, or imagining connections may be an important aspect of recovery-oriented practice in forensic psychiatric care.

## Data Availability

The datasets presented in this article are not readily available due to the sensitive and potentially identifiable nature of qualitative data. Requests to access the datasets should be directed to berini@rm.dk.
